# Efficacy of Fungi in the Decolorization and Detoxification of Remazol Brilliant Blue Dye in Aquatic Environments

**DOI:** 10.3390/microorganisms11030703

**Published:** 2023-03-09

**Authors:** Nabeela, Sumera Afzal Khan, Saadat Mehmood, Sohaib Bin Shabbir, Sajid Ali, Abdulwahed Fahad Alrefaei, Mohammed Fahad Albeshr, Muhammad Hamayun

**Affiliations:** 1 Centre of Biotechnology and Microbiology, University of Peshawar, Peshawar 25120, Pakistan; 2 Department of Horticulture and Life Science, Yeungnam University, Gyeongsan 38541, Republic of Korea; 3 Department of Zoology, College of Science, King Saud University, Riyadh P.O. Box 2455, Saudi Arabia; 4 Department of Botany, Garden Campus, Abdul Wali Khan University Mardan, Mardan 23200, Pakistan

**Keywords:** fungal isolates, blue dye, decolorization, toxicity test, seed germination, environmental pollution

## Abstract

Industrial effluents result in water pollution and affect the biological activity of aquatic and terrestrial life. In this study, efficient fungal strains were isolated from the aquatic environment and identified as *Aspergillus fumigatus* (SN8c) and *Aspergillus terreus* (SN40b). The isolates were selected based on their potential to efficiently decolorize and detoxify Remazol brilliant blue (RBB) dye, which is extensively used in different industries. Initially, a total of 70 different fungal isolates were screened. Among these, 19 isolates demonstrated dye decolorization capabilities, and SN8c and SN40b revealed the highest decolorization capabilities in liquid medium. The maximum estimated decolorization for SN8c was 91.3% and for SN40b, 84.5% at 40 mg/L of RBB dye in the presence of glucose (1 gm/L), after 5 days of incubation at different levels of pH, temperature, nutrient source, and concentration. RBB dye decolorization using SN8c and SN40b isolates was at a maximum of 99% at pH 3–5, whereas minimum decolorization was recorded as 71.29% and 73.4% SN8c, respectively, at pH 11. The maximum decolorization of the dye was 93% and 90.9% in a defined glucose concentration of 1 gm/L, and a 63.01% decrease was recorded in the decolorization activity at a low level of glucose concentration (0.2 gm/L). Finally, the decolorization and degradation were detected using UV spectrometry and HPLC. Toxicity tests of pure dye and treated dye samples were checked against the seed germination of different plants and the larvae mortality of *Artemia salina*. This study revealed that indigenous aquatic fungal flora can recover contaminated sites and support aquatic and terrestrial life.

## 1. Introduction

Various types of synthetic organic colorants are used as dyes in different industries ranging from textile production to pharmaceutical, food, cosmetics, printing, leather, and textile industries [1,2,3]. Reactive dyes are being increasingly used in dyeing procedures because of their greater solubility, stability, resistance to microbial deterioration, wash-fastness, and stronghold coloring properties [4,5]. The actual volume of dye produced worldwide is unknown, although it is expected to be over 10,000 tons per annum, with 10 to 15% of synthetic dyes discharged during various industrial processes [6,7,8]. Dyes such as Remazol brilliant blue (RBB) are industrially valuable and can be used as a starting material in the production of polymeric dyes; however, recent reports revealed RBB as a class of toxic and recalcitrant organic pollutant [9]. The dye effluents discharged from textile industries contain reactive dyes and greater amounts of NaCl contents that pose a high risk to natural environmental conditions [10]. During dyeing procedures, about 10–40% of the dyestuffs enter the aquatic environment, leading to serious environmental problems [1]. Thus, the prior detoxification of dye before it enters the natural environment is pivotally important.

Previously, researchers have suggested a variety of different techniques, including filtration, adsorption, chemical transformation, and precipitation, for the remediation of dye effluents from various main processes in different industries [11]. Recent studies have revealed different types of fungal (*Aspergillus*, *Alternaria*, *Cladosporium*, *Penicillium*) and bacterial species (*Bacillus subtilis*, *Escherichia coli*, *Pseudomonas*, etc.) that produce extracellular enzymes and can biodegrade complex organic compounds through catalysis and play important role in the detoxification and decolorization of dye effluents, mitigating their adverse effects on the natural ecosystem [10,12,13,14,15]. More recently, various fungal species have been reported for their production of extracellular ligninolytic enzymes such as lignin and manganese peroxidases, and lactase, which also perform a prominent role in the biodegradation of organic compounds [10,16,17]. The contamination of these synthetic organic compounds in wastewater has an adverse effect on the biological activity of aquatic life by limiting the availability of light and causing toxicity in the ecosystem.

Generally, dyes are colored compounds that absorb light of a wavelength between 400 and 700 nm [18]. Based on their chemical structure, the organic dyes used in the textile industry are classified as azo, diazo, cationic, basic, and anthraquinone base with unique characteristics [17]. The sulfonic acid group of anthraquinone dye is responsible for its water solubility and is frequently used in the food and textile industries. The most important class of dye used in the textile industry is RBB, which is typically applied as an early material in the production of polymeric dyes [19]. However, RBB is an anthracene derivative that describes an important group of hazardous pollutants. The chemical and physical approaches for treating wastewater at textile manufacturing sites have drawbacks, such as disposal issues, high cost, and low effectiveness [6,20].

On the other hand, biological methods are highly effective and environmentally favorable due to the complete mineralization of carbon-based contaminants. This makes biological treatments more attractive compared to physical and chemical strategies. More recently, various studies revealed the application of competent fungi in the treatment of wastewater. Fungal species producing extracellular enzymes, such as lignin peroxidases, (Lip) manganese peroxidases (MnP), and lactases, revealed their capabilities in oxidizing different organic pollutants containing artificial dyes. Similarly, biological mechanisms such as bioaccumulation, biodegradation, and biosorption increase the attractiveness of using fungal species in the removal of pollutants [1,21,22].

The current studies on the biodegradation and decolorization of dyes using different fungi show that it is a low-budget, highly effective, and eco-friendly practice, and a very significant tool for poisonous compounds such as (textile and tannery wastes). Subsequently, if the degradation frequency is remarkable, the concentration, and in this manner, the noxious effect, will be abridged expeditiously, although very persistent chemicals will maintain their toxicity for a very long time.

Thus, the search for competent fungal species to degrade industrial dyes and reclaim contaminated soils is of great importance. In this study, we aimed to isolate, identify and characterize the role of aquatic fungal species in the decolorization and detoxification of Remazol brilliant blue (RBB) dye. The main objectives of this study are to optimize the growth conditions for the biodegradation of Remazol brilliant blue dye and to evaluate the toxic effect of the dye against selected seeds and aquatic crustaceans.

## 2. Materials and Methods 

### 2.1. Chemicals

Dyes and chemical compounds used in the research work were extrapure and of HPLC grade, obtained from Duksan Pure Chemicals, Republic of Korea, Honeywell France, and Merck KGaA, 64271 Darmstadt, Germany.

### 2.2. Sampling Site and Isolation of Fungi

Dye-contaminated water and sludge samples were collected in sterilized bottles from the wastewater tanks of Saif textile mills in the Gadoon Amazai area of Swabi’s industrial sector. The samples were taken to the Microbiology Research Laboratory (MRL), Center of Biotechnology and Microbiology (COBAM), University of Peshawar, Khyber Pakhtunkhwa, Pakistan. The serial dilution technique was used to isolate fungi from the contaminated sludge samples according to the detailed methods of [17,23,24]. Briefly, 10 gm of each sample was dissolved in 90 mL of sterile distilled water and serially diluted. Then, each diluted sample (1 mL) was equally spread on potato dextrose agar medium plates supplemented with penicillin to prevent bacterial contamination and incubated at 28 °C for 7 days consecutively [25]. After seven days of incubation, the colonies were screened and isolated on PDA supplemented with penicillin to prevent bacterial contamination and examined microscopically for the identification of pure culture according to the detailed methods of Zhiqiang An [26]. Identified isolates were preserved at 4 °C on PDA slants at the Microbiology Research Laboratory (MRL), Center of Biotechnology and Microbiology (COBAM), University of Peshawar, Khyber Pakhtunkhwa, Pakistan.

### 2.3. Screening of Fungi for Decolorization of RBB Dye and Decolorization Analysis

The pure colonies of fungal isolates were screened for the decolorization of RBB dye using the detailed method of Ramya, et al. [27]. PDA plates with different concentrations of RBB dye (50, 100, 150, 200, 250, 300, 350, 400, 450, and 500 mg) were evaluated for the decolorization of the dye. Similarly, a liquid medium (broth) was also used to compare the activity of the fungal isolates in solid and liquid media. In a liquid medium, different concentrations (40–100 mg) of the dye were used in a shaking incubator at 120 rpm, 28 ± 2 °C for 7 days [23]. Decolorization analyses were performed noticing the results of optical density using a UV spectrometer (Perkin-Elmer). To measure the decolorization rate more precisely, 2 mL of each sample was filtered after each 24 h and centrifuged; the supernatant was isolated and analyzed at a wavelength of 590 nm using the UV spectrophotometer. All of the experiments were carried out in triplicate and the decolorization efficiency was measured using the following formula:

*Decolorization* (%) = [(*InitialAbsorb.* − *FinalAbsorb.*)/*InitialAbsorb.*] × 100

### 2.4. Optimization of Growth Condition for Augmented Decolorization

The chemical and physical conditions were optimized for higher decolorization. The physiochemical parameters including dye and glucose concentrations, carbon source, and the temperature and pH of the fungal isolates were varied for optimum decolorization of the dye. Mycelia (with a 10 mm agar piece) of selected fungal isolates were inoculated into the flasks containing the required media supplemented with RBB dyes. The flasks were incubated in a shaking incubator (120 rpm) at 28 ± 2 °C for 120 h. The decolorization rate of the dye was analyzed using a UV spectrophotometer every 24 h for five consecutive days for all parameters, according to the detailed method of Ponraj, Jamunarani, and Zambare [17].

### 2.5. Effect of pH, Temperature, and Dye Concentration

The fungal isolates were evaluated for their ability to decolorize RBB at a pH of 3, 5, 7, 9, and 11. Similarly, the effect of different temperatures (25 to 50 °C) on dye decolorization was determined using actively growing fungal mycelia according to the detailed method of Khan, Mehmood, Iqbal, Hamayun, and Safety [10]. The percentage of decolorization was evaluated at various concentrations (40–100 ppm) of RBB dye. Briefly, 10 mm of the fungal mycelia was added to a flask containing media under sterile conditions and kept in a shaking incubator for 120 h at 28 ± 2 °C. The method of Sathishkumar, Balan, Palvannan, Kamala-Kannan, Oh, and Rodríguez-Couto [2] was followed for the evaluation of the decolorization rate using a UV spectrophotometer.

### 2.6. Effect of Glucose Concentration and Carbon Sources

The decolorization of RBB dye was also evaluated at various concentrations (0.2, 0.4, 0.6, 0.8, and 1 g) of glucose using fungal isolates. The detailed method of Khan, Mehmood, Iqbal, Hamayun, and Safety [10] was followed for investigating the effect of additional carbon sources such as lactose, fructose, sucrose, and glucose (extrapure grade, Duksan, Korea) on dye decolorization using the fungal isolates.

### 2.7. Analysis of Biodegradation 

For the evaluation of the biodegradabilities of the products, high-performance liquid chromatography (Shimadzu HPLC) was used. The detailed method of Lade, et al. [28] was followed for the biodegradation analysis. Briefly, HPLC analysis was carried out on a C-_18_ column (250 × 4.6 mm) with Methanol:Acetonitrile (1:1) (99.9% HPLC grade, Honeywell, France) as a mobile phase in the HPLC with a flow rate of 1.0 mL/min and UV detection at 590 nm.

### 2.8. Enzymatic Assay (Manganese Peroxidase)

After their successful function in the decolorization, the prospective fungal isolates were evaluated for manganese peroxidase activity in Bushnell Hass Medium (BHM). A 0.5 mL Guaiacol (extrapure grade, Duksan, Republic of Korea) (0.1 M) was mixed with 0.5 mL of 0.1 M Sodium tartrate buffer (extrapure grade, Duksan, Republic of Korea) (pH-5.0), 0.5 mL of 0.1 M MnSO_4_, and 0.05 mL of H_2_O_2_. The availability of peroxidase enzymes significantly oxidizes the guaiacol into tetraguaiacol, which results in a reddish-brown color after a successful reaction. In this way, the activity of manganese peroxidase was evaluated by measuring the color absorbance at 465 nm after guaiacol was oxidized. The methods of Srinu, et al. [29] were followed for the calculation of peroxidase activity.

### 2.9. Toxicity Testing of RBB (Direct Dye and Treated Dye Products)

#### 2.9.1. Seed Germination Inhibition Activity

The seeds of different plants (melon, lentil, wheat, and watermelon) were thoroughly washed with distilled water and placed on filter paper at the bottom of Petri plates. The filter paper was treated with RBB dye (5 mL) at various concentrations ranging from 50 to 250 mg. The method of Rathod, et al. [30] was followed to evaluate the effect of the dye on seed germination. Each experiment was repeated in triplicate, while data collected from each treatment and repeated experiment were compiled for further analysis.

#### 2.9.2. Aquatic Animal Toxicity Test

The toxicity effect of RBB dye was also evaluated on an aquatic animal. *Artemia salina* (brine shrimp) was used as an experimental animal to check the level of toxicity of RBB dye. The cysts of *A. salina* were placed in seawater for 24 h at room temperature until hatched. After the successful hatching of the cysts, viable larvae were subjected to different concentrations of the dye such as 10, 100, and 1000 μg/mL. Similarly, 15 *A. salina* larvae were placed in vials and incubated at room temperature for 24 h. The method of Sani, et al. [31] was followed for the calculation of the mortality rate.

Formula (2) for calculating the mortality rate:

Mmct = Nmm/No. × 100

where Mmct is the mortality of individuals in time, t (%), Nmm is the average number of dead individuals, and No is the initial number of living individuals subjected to each concentration.

### 2.10. Molecular Identification and Phylogenetic Analysis of the Fungal Isolates

The detailed methods of [10,32] were followed for the molecular identification and phylogenetic analysis of selected fungal isolates, with slight modifications. Briefly, the genomic DNA was extracted using a fungal DNA extraction kit (Norgen Biotek Corp., product No. E5038) and a spectrophotometer (Thermo-Scientific, Wilmington, DE, USA) was used to analyze the quantity and purity of the extracted DNA at 260 nm. The isolated DNA was amplified through PCR by amplifying the ITS region of 18S rDNA with universal primers (ITS-1:5′-TCCGTAGGTGAACCTGCGG-3′) and (ITS-4:5′-TCCTCCGCTTATTGATATGC-3′). Genomic DNA (20 ng) as a template was used in a 30 μL reaction mixture along with Tag (Solgent, Daejeon, Republic of Korea) for the PCR reaction. The complete reaction was as follows: denaturation at 95 °C for 2 min, 35 cycles (95 °C for 60 s, 55 °C for 60 s, and 72 °C for 60 s), and then 72 °C for 10 min. The marker DNA (a DNA ladder) and PCR product were loaded onto an agarose gel and subjected to electrophoresis for 30 min. Finally, the gel was stained with ethidium bromide (0.01 g/mL) and examined under a UV transilluminator lamp. Identical nucleotide sequences of the ITS region were compared using the NCBI-BLAST search engine, and the sequences were utilized to generate a phylogenetic tree using the maximum-likelihood approach in MEGA-7 software [33,34,35].

### 2.11. HPLC Analysis of Dye Degradation

The two fungi, SN8c and SN40b, after decolorization activities, were first filtered through Whatman filter paper No. 1 and then centrifuged for 10 min at 10,000 rpm in each flask to study the biodegradation activity. The supernatant was passed through a 0.2 μm syringe filter and the biodegraded products were analyzed using a Shimadzu HPLC at 590 nm using a UV–VIS detector with a C18 column (symmetry, 250 4.6 mm). HPLC-grade methanol and acetonitrile (1:1) were used as the mobile phase, according to the detailed methods in [10,36].

### 2.12. Statistical Analysis

Data collected from each treatment and repeated experiments were compiled for further analysis. All experiments were performed in triplicate and expressed as a mean value using Duncan’s multiple range test (DMRT).

## 3. Results

### 3.1. Fungal Isolation and Evaluation for Dye Decolorization

**Figure 1:**
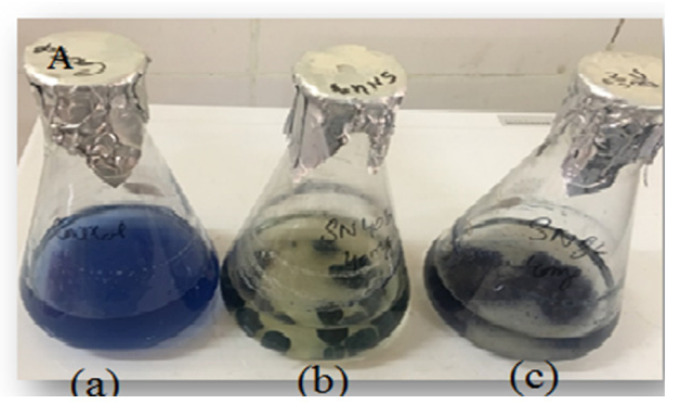
The decolorization of RBB dye in liquid medium: (**a**) control, (**b**) SN40b, and (**c**) SN8c.

In the present study, a total of 70 fungal isolates from the sludge of the textile industry at Gadoon Amazai Industrial Estate in the district of Swabi, Pakistan. The isolated fungal species were evaluated for their potential decolorization of RBB dye on dye-amended solid and liquid media. Out of seventy fungal species, 19 were found to be competent in the decolorization of the liquid medium, whereas twenty species revealed decolorization activities in the solid medium. However, two fungal isolates (SN8c and SN40b) were specifically selected for further studies because of their significant decolorization capability, as shown in Figure 1.

### 3.2. Optimization of Physical and Chemical Parameters for Fungal Growth and Decolorization

The physiochemical parameters including RBB dye and glucose concentrations, pH, temperature, and source of carbohydrates were varied for the fungal isolates (SN8c and SN40b) that showed prominent results in initial screening for decolorization. The rate of dye decolorization was estimated using a UV spectrophotometer after an interval of 24 h for 5 days consecutively.

### 3.3. Effect of RBB Dye Concentration on Decolorization

At a concentration of 40 mg/L, the SN8c and SN40b fungal isolates decolorized RBB dye by 91.3% and 84.5%, respectively. However, the lowest decolorization value for RBB dye was observed at the concentration of 100 mg/L for SN8c (11.35%) followed by SN40b (8.70%) when the decolorization activity was calculated after 5 days of fungal incubation (Table 1).

### 3.4. Effect of pH on Decolorization of RBB Dye

**Figure 2:**
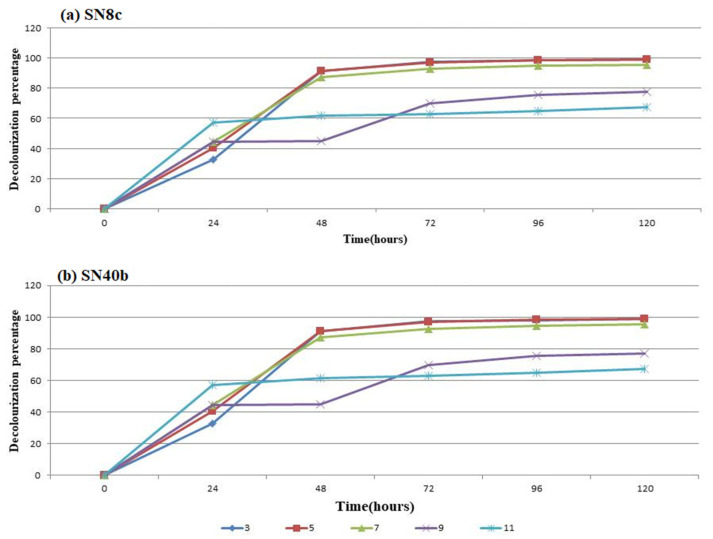
The effect of pH on RBB dye decolorization for (**a**) SN8c, and (**b**) SN40b.

The decolorization of RBB dye using the SN8c and SN40b selected fungal strains was estimated after 5 days and it was observed that SN8c and SN40b decolorized more than 85% of RBB dye at a lower pH (~5) and neutral pH (~7) after 48 h. On the other hand, the activity was very low after 24 h, decreased to 70% after 48 h at a higher pH (~11), and remained constant over time (Figure 2).

### 3.5. Temperature Effect on the Decolorization of RBB Dye

**Figure 3:**
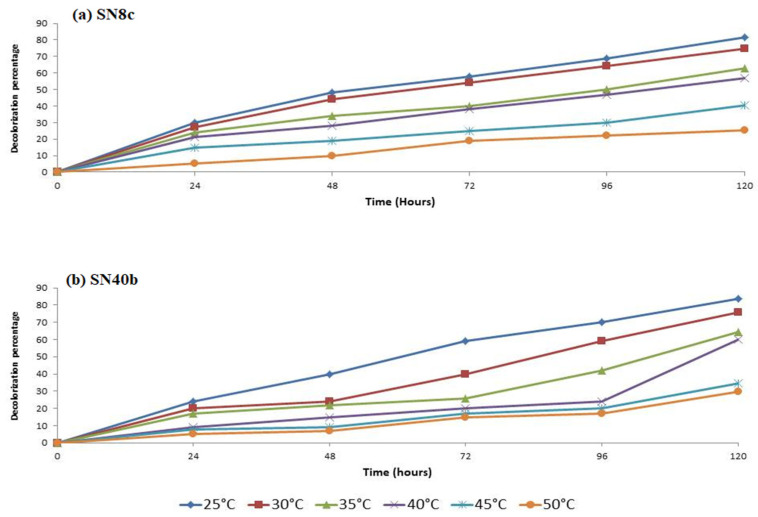
The effect of temperature on the decolorization of RBB dye.

The effect of temperature on the decolorization of RBB dye using selected fungal isolates was determined under different temperature conditions ranging from 25 °C to 50 °C (Figure 3). More than 80% of RBB dye decolorization was observed for both fungal isolates (SN8c: 83% and SN40b: 81.7%) after 5 days of incubation at 25 °C. On the other hand, the decolorization of the dye was significantly less at 50 °C after 5 days; up to 25% less for SN40b and up to 30% less for SN8c.

### 3.6. Effect of Different Glucose Concentrations on the Decolorization of RBB Dye

**Figure 4:**
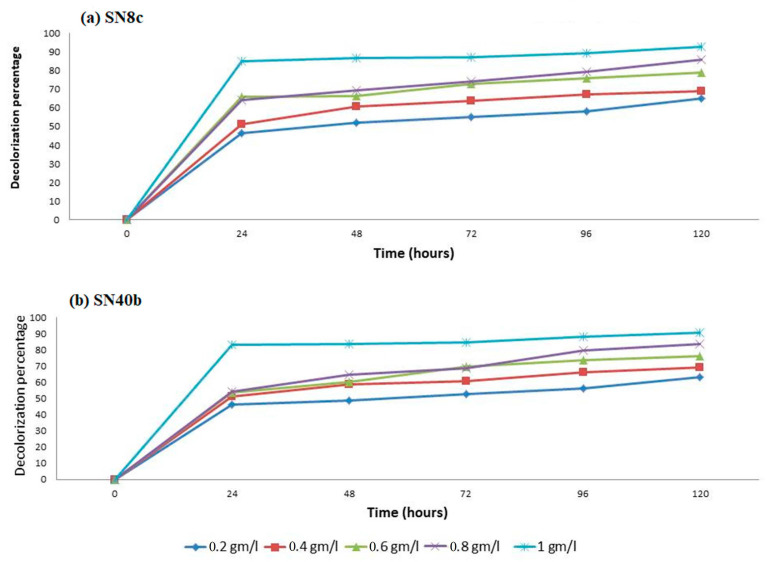
The effect of different glucose concentrations on the decolorization of RBB dye.

The influence of the available glucose in the media on RBB dye decolorization was studied. Our study revealed that fluctuating or changing the amount of glucose concentration in the media from 0.2 g/L to 1 g/L significantly affected the decolorization capability of both fungal isolates. The maximum decolorization of the RBB dye using SN8c and SN40b was 93% and 90.9% in 1 g/L of glucose. However, both fungal strains revealed a minimum level of dye decolorization in a low media glucose concentration of 0.2 g/L (Figure 4).

### 3.7. Effect of Carbohydrate Source on the Decolorization of the Dye

**Figure 5:**
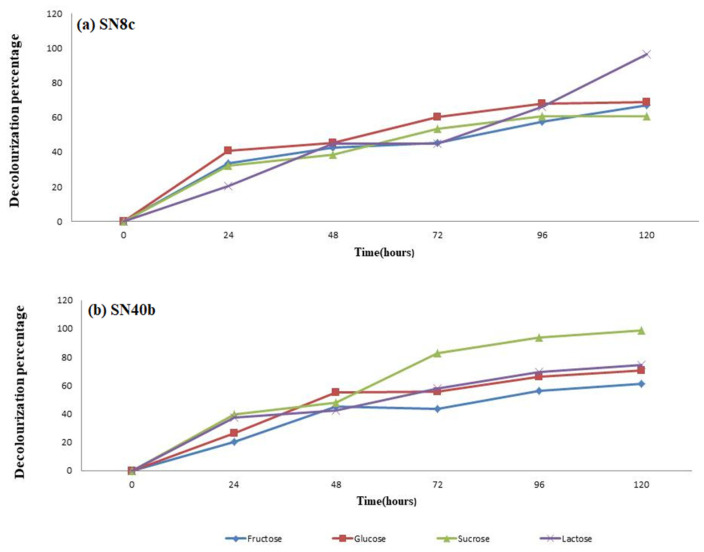
The effect of different carbohydrate sources on the decolorization of RBB dye.

The effect of different carbohydrate sources was investigated by providing equal amounts of fructose, glucose, sucrose, and lactose in the media (Figure 5). The maximum decolorization of RBB dye using inoculated fungal strains (SN8c and SN40b) was observed in the media supplemented with lactose and sucrose. The fungal strain SN40b showed 93.58% decolorization in the presence of sucrose, whereas SN8c revealed 96.23% decolorization in the presence of lactose. The lowest decolorization activity in the media containing fructose was 56% and 67% for SN40b and SN8c, respectively.

### 3.8. Biodegradation Analysis of Remazol Brilliant Blue by HPLC

**Figure 6:**
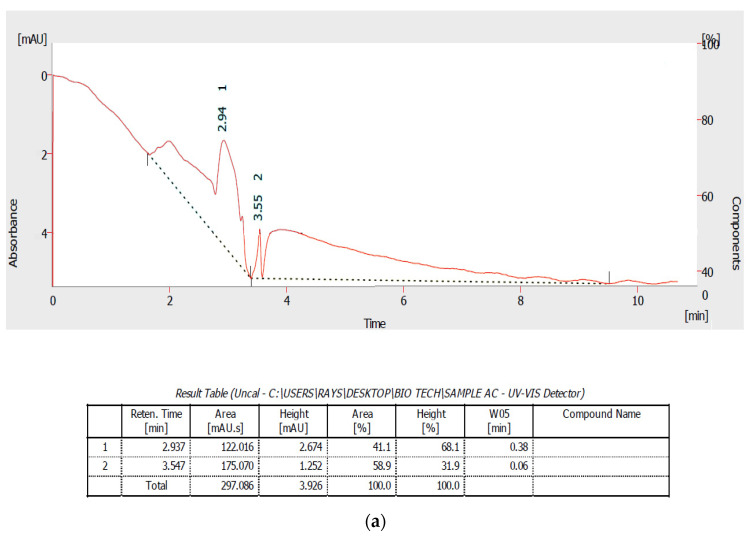

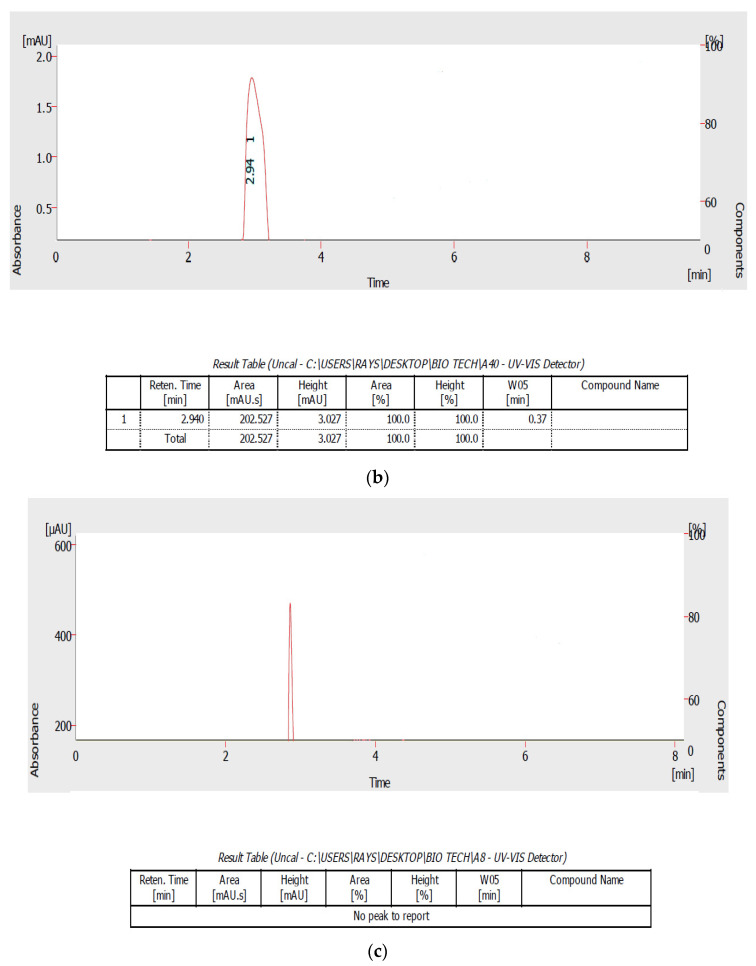
(**a**) Remazol brilliant blue control. (**b**) HPLC profile of RBB (RBBR) degradation by *A. terreus.* (**c**) HPLC profile of RBB (RBBR) degradation by *A. fumigatus*.

The HPLC analysis of the Remazol brilliant blue control showed the presence of two major peaks at a retention time of 2.93 and 3.54 min (Figure 6a). After the dye decolorization process using SN40b, a disappearance of peaks in the control and the formation of completely different peaks at retention times of 2.94 min was observed (Figure 6b). The decolorization of the dye using SN8c showed the complete disappearance of the major peaks observed in the control chromatogram. The appearance of new minor peaks and the disappearance of the major peak in the decolorized dye product elution profile support the biodegradation results of Remazol brilliant blue by these fungal species (Figure 6c).

### 3.9. Enzymatic Activity (Manganese Peroxidase) by Fungal Isolates

**Figure 7:**
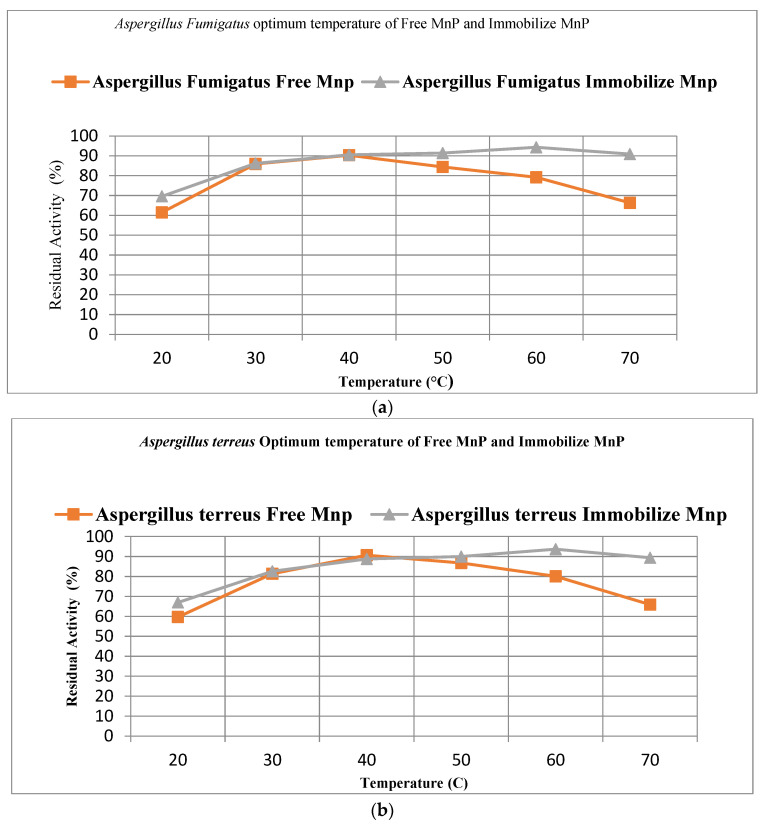
(**a**) The optimum temperatures for free and immobilized MnP from SN8c. (**b**) The optimum temperatures for free and immobilized MnP from SN40b.

After their effective role in the decolorization process, the potential fungal isolates were evaluated for manganese peroxidase activity in Bushnell Hass Medium (BHM). Manganese peroxidase enzymatic activity was measured using guaiacol as a substrate. The ultimate change in the color showed the manganese peroxidation activity of SN8c and SN40b. The selected fungal strain SN8c exhibited a higher manganese peroxidation activity (0.4959 U/mL) compared to SN40b (0.3719 U/mL) (Figure 7).

#### 3.9.1. Optimum Temperature

Free and agar–agar-entrapped MnP from both isolates A. fumigatus and A. terreus show maximum relative activities at 40 °C and 60 °C, respectively, with 90.3% and 94.5% from A. fumigatus and 90.7% and 93.7% for A. terreus. Optimum temperatures for both isolates elevated from 40 °C to 60 °C due to immobilization, which confirms that immobilized enzymes are thermally more stable than free enzymes. Free enzymes from A, fumigatus show 61.5% and 85.9% activity at 20 °C and 30 °C while achieving maximum activity at 40 °C. Further assessing the activity at 50 °C, 60 °C, and 70 °C, the activity declines with 84.4%, 79.2%, and 66.3%, whereas agar–agar-entrapped MnP from A. fumigatus shows 69.6%, 86.2%, 90.5%, and 91.4% activity at 20 °C, 30 °C, 40 °C and 50 °C, further achieving maximum activity at 60 °C. At 70 °C, the activity declines to 90.9%. Free enzymes from A. terreus showed 59.7% and 81.4% activity at 20 °C and 30 °C, while at 50 °C, 60 °C and 70 °C, activities were recorded 86.8%, 80.1%, and 65.9%, respectively. Agar–agar-entrapped MnP was recorded to have 67%, 82.6%, 88.8%, and 90% activities at 20 °C, 30 °C, 40 °C, and 50 °C, achieving maximum activity at 60 °C, and showing a decline at 70 °C with 89.4% activity, as shown in Figure 7a,b, respectively.

#### 3.9.2. Temperature Stability

**Figure 8:**
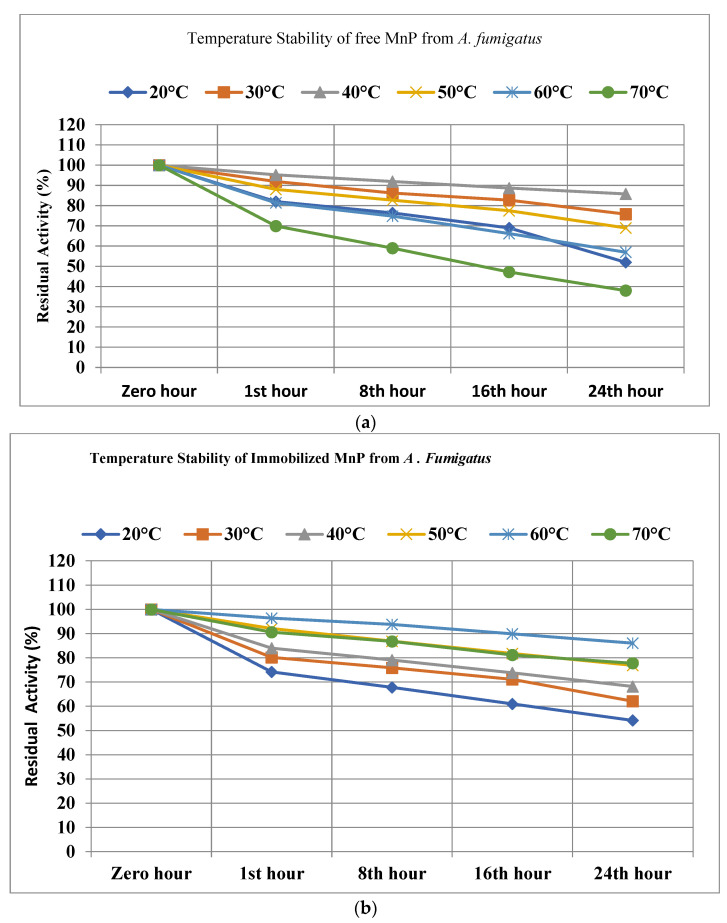
(**a**) Temperature stability of free MnP from *A. fumigatus.* (**b**) Temperature stability of immobilized MnP from *A. fumigatus*.

Temperature is considered an important factor while dealing with immobilized enzymes, and the thermostabilities of immobilized and free MnP were investigated by incubating at different temperatures (ranging from 20 °C to 70 °C), and the residual activity was noted at the indicated time. Agar–agar-entrapped MnP from *A. fumigatus* were noted to have the highest thermostability compared to its free enzymes, with 86.1% activity at 60 °C and 77.8% at 70 °C, while free enzymes resulted in 57% and 38% activity, respectively. Further, at 20 °C, 30 °C, 40 °C, and 50 °C, the entrapped MnP resulted in 54.2%, 62.1%, 68.2%, and 76.9% activity, while free MnP was noted to have activities of 52%, 75.8%, 85.8%, and 69%, respectively (Figure 8a,b).

**Figure 9:**
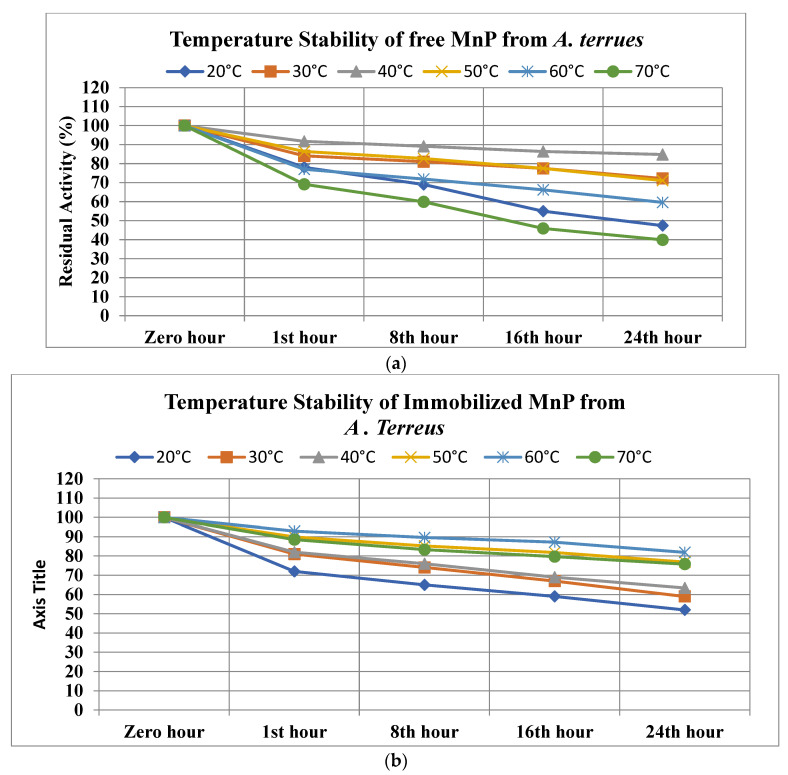
(**a**) Temperature stability of free MnP from *A. terreus.* (**b**) Temperature stability of immobilized MnP from *A. Terreus*.

Whereas entrapped MnP from A. terreus resulted in 81.9% at 60 °C and 75.8% at 70 °C while free MnP showed 59.6% and 39.9%, respectively, at 20 °C, 30 °C, 40 °C and 50 °C, agar–agar-entrapped MnP showed 52%, 59%, 63.4%, and 76.9% residual activity, and free MnP resulted in 47.4%, 72.2%, 84.8%, and 71.1% activity (Figure 9).

### 3.10. RBB Dye Toxicity Test (Pure and Treated Dye)

#### 3.10.1. Seed Germination Inhibition Activity

The seeds of various crop plants were soaked in RBB dye to evaluate the effect of both direct and treated dye on the germination capability of the seeds. Surprisingly, the maximum seed germination (100%) was recorded in all samples of the treated dye except watermelon (60%). On the other hand, seed germination activity was greatly reduced (0%) in all samples treated with a higher concentration of pure RBB dye (250 mg) (Table 2).

#### 3.10.2. Toxicity Testing of RBB Dye against *Artemia salina*

*A. salina* was used to perform a toxicity test of RBB dye. Different concentrations of RBB dye and textile industry effluents were used to evaluate the mortality rate of *A. salina* larvae. After 24 h, the mortality rate was 100% at a concentration of 1000 μg/mL. On the other hand, the fungal-treated samples revealed a low level of toxicity and the mortality rate was reduced to 6% in the case of SN40b and 13% in the case of SN8c at a concentration of 1000 μg/mL (Table 3).

### 3.11. DNA Extraction and Molecular Identification 

**Figure 10:**
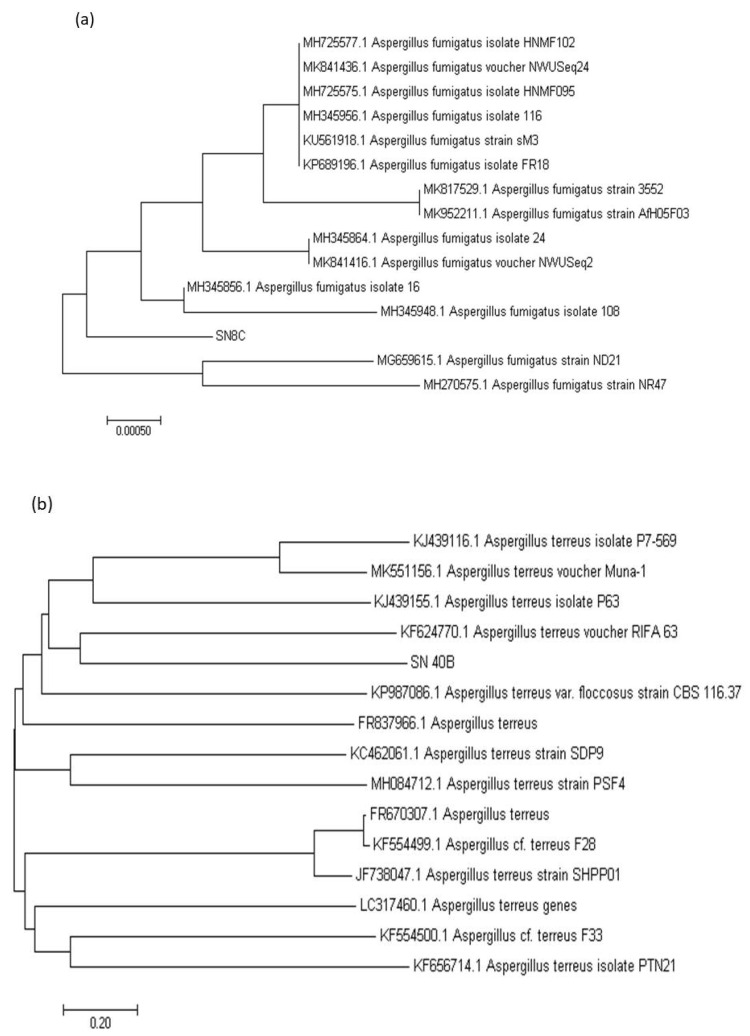
(**a**,**b**) Phylogenetic tree constructed through the maximum parsimony method using MEGA 6.0 [37]. The sequence obtained from ITS regions of rDNA of isolated fungi were making good clads with fungi sequences from BLAST. (**a**) The phylogenetic tree of SN8c identified as *A. fumigatus*, and (**b**) the phylogenetic tree of SN40b identified as *A. terreus*.

The sequences were obtained through Macrogen, Korea, and the ITS regions of genomic DNA of fungal isolates were used for their molecular identification. The sequences revealed more than 99% homology with different species of *Aspergillus* and phylogenetic analysis confirmed our results. The sequences of the fungal isolates SN8c and SN40b were evaluated for their alignment with allied sequences and highly related sequences were downloaded using MEGA7 software. BLAST search program and phylogenetic tree results showed that SN8c has 99.9% similarity with *Aspergillus fumigatus,* while SN40b has 100% similarity with *Aspergillus terreus* (Figure 10a,b).

## 4. Discussion

The application of microorganisms is preferable to physical and chemical approaches for the decolorization and detoxification of industrial effluents because the use of microorganisms to remove pollution from the environment is a cost-effective and environmentally-friendly approach. The wastewater discharged by different industries including textile and food contains a higher concentration of dyes, which greatly affect the biological activity of terrestrial and aquatic life. In this study, 70 fungal isolates were obtained from textile industry effluents and their decolorization and detoxification abilities were evaluated over a range of RBB dye concentrations and physical and chemical conditions. Nineteen of the 70 fungal isolates tested proved to have strong decoloring activities against RBB dye. Similar studies by Khan, Mehmood, Iqbal, Hamayun, and Safety [10] were followed, where they reported the isolation of 86 fungal isolates and evaluated 31 potent fungal species decolorizing Synozol Red and Synozol Black dye. In another study, researchers from Mulawarman University, Indonesia, reported a total of 24 fungal species. The capacity of 24 fungal species to decolorize was tested on an agar plate treated with RBB R and cultured for 5 days. Out of 24 fungal isolates, 9 strains showed decolorization results [38].

This study revealed that the fungal strains SN40b and SN8c have RBB dye decolorization capabilities of up to 95% after five days of treatment. In this regard, Afiya, et al. [39] reported the decolorization capabilities of *Pleurotus ostreatus* and *Coriolus versicolor* of up to 70% and 80%, respectively, against RBB dye in eight days of treatment. The strains evaluated in this study showed better results than the previously reported fungal isolates. Moreover, physiochemical parameters greatly affect the decolorization capability of microorganisms including pH, temperature, dye concentration, carbon source, and other essential nutrients. The dye concentration plays a pivotal role in the decolorization of the dye when using microbes. The inoculation of Aspergillus fumigatus (SN8c) and Aspergillus terreus (SN40b) revealed maximum decolorizations of up to 77% and 86% at a concentration of 40 mg/L, respectively. However, the capacity of decolorization was reduced by up to 60% at an RBB dye concentration of 100 mg/L. Our findings are consistent with the study of Ramya, Anusha, Kalavathy, and Devilaksmi [27], in which they reported the decolorization of Acid-Red 151 by using *A. flavus* SA2 (93%), *A. terreus* SA3 (88%) and *A. niger* SA1 (82%) at a low concentration (50 mg/L).

Carbon sources such as glucose, lactose, sucrose, and fructose are used by the majority of fungi [40]. *A. terreus* (SN40b) showed the highest decolorization of RBB dye (93.58%) in media containing sucrose, while *A. fumigatus* (SN8c) showed the lowest (69%) in media containing glucose. Our findings were in line with previous research in which glucose and starch were utilized as carbon sources to decolorize Reactive Black B (90%) and Reactive Orange (16.85%) in media [41]. Moreover, different pH values revealed optimized conditions for the fungal strains to decolorize RBB dye. At a pH of 3, 5, and 7, *A. fumigatus* (SN8c) and *A. terreus* (SN40b) decolorized RBB dye to a maximum of 99%; however, at pH 9 and 11, the activity was reduced to 70%. The decolorization activity of textile dye T. Blue through *A. flavus* was 93% at a pH of 4, similar to the current investigation, while the activity of *A. niger* increased to 97% at a pH of 6 [17]. Similarly, temperature plays a vital role in the decolorization activity of fungal isolates. Various temperature conditions (25 to 50 °C) were used in this study to find the optimal conditions for the decolorization of RBB dye utilizing fungal isolates. At 25 °C, *A. fumigatus* (SN8c) and *A. terreus* (SN40b) removed 83% of the RBB dye; however, the rate of decolorization decreased to 25% as the temperature rose to 50 °C. Similarly, at 37 °C, *A. flavus* and *Helminthosporium* sp. decolorized RBB dye by 95% and 75%, respectively, indicating that the biosorption capability declines as temperature rises [42]. Furthermore, the manganese peroxidase activity of *A. fumigatus* (SN8c) and *A. terreus* (SN40b) was measured at 0.04959 U/mL and 0.03719 U/mL after 12 days of incubation using Bushnell Haas Medium. Similarly, the *A. fumigatus* strain YHYS had the highest enzymatic activity of 33.9 U/mL, whereas the *A. tamarii* strain SRRC 1088 had the highest enzymatic activity of 287.6 U/mL [29]. 

The toxicity of the RBB dye was investigated in this study utilizing the seed germination capability of five different plants. The germination percentage of these seeds was 80–100 percent when the RBB dye concentration was 50–100 mg/L; however, when the dye concentration was increased to 250 mg/L, the germination percentage dropped to 10–40 percent. Already reported studies have revealed the effects of synthetic dyes and their changing concentrations on the germination of seeds of various plant species, including *T. aestivum*, *V. radiata*, *V. aconitifolia*, *V. sinensis*, and *C. arietinum*, and it showed that the germination percentage of all plants was completely inhibited as the dye concentration increased from 3000–90,000 ppm [42,43]. Moreover, toxicity tests of RBB dye, real textile effluents, and treated products of fungal species were performed on *A. salina*. When the concentrations of RBB dye and genuine textile effluents were increased from 10 g/mL to 1000 g/mL, the death rate increased by 50–100%. Because fungal effluents are innocuous, the mortality rate was lowered by up to 15% when they were used. Previous studies on *A. salina* included blue, orange, red, and yellow dyes, which are routinely used in the dyeing process. All of these studies reported that blue dye has a mean percentage mortality of 100%, yellow dye has a percentage mortality of 90%, red dye has a mortality rate of roughly 60%, and orange dye has a percentage mortality rate of 47%, making it the least poisonous of the four dyes studied [31]. In this study, toxicity was determined by administering small amounts of material to a limited number of *A. salina* and by observing the dose-response relationship for a set length of time. However, the toxicity of the dye is reduced by using competent fungal strains.

## 5. Conclusions

The degradation of toxic Remazol brilliant blue (RBB) dye by *Aspergillus fumigatus* (SN8c) and *Aspergillus terreus* was revealed in a recent study. The novelty of the present study is based on the complete decolorization under the influence of different environmental factors including dye concentration, temperature, glucose concentration, carbohydrates sources, and acidic pH (3–5), facilitated by extracellular enzymes cleaving the RBB dye. Finally, our findings highlight the relevance of dye decolorization using indigenous fungal flora at the discharge site of industrial effluents for the efficient removal of dye pollutants from our natural environment. Thus, *A. fumigatus* (SN8c) and *A. terreus* (40b) are potential bioremediating tools for wastewater treatment in contaminated sites with anthraquinone dyes.

## Figures and Tables

**Table 1:** Effect of RBB dye concentration on decolorization.

Dye Concentration	Fungal Isolate SN8c	Fungal Isolate SN40b
40 mg/L	91.3%	84.5%
50 mg/L	80.79%	75%
60 mg/L	62.5%	58.7%
70 mg/L	54.32%	49.80%
80 mg/L	38.60%	35.26%
90 mg/L	22.6%	19.45%
100 mg/L	11.35%	8.70%

**Table 2:** Seed germination inhibition activity.

Seeds	Germination Percentage Against Pure Dye and Treated Dye Sample (SN8C and SN40b)						
	50 mg	100 mg	150 mg	200 mg	250 mg	SN8C	SN40
Lentil	100 ± 2.01	100 ± 1.75	40 ± 1.26	20 ± 0.32	0 ± 0.03	100 ± 2.13	100 ± 2.48
Wheat	60 ± 1.24	60 ± 1.04	40 ± 1.12	20 ± 0.25	0 ± 0.04	100 ± 2.09	100 ± 2.13
Watermelon	20 ± 0.13	20 ± 0.47	20 ± 1.41	0 ± 0.01	0 ± 0.00	60 ± 1.89	80 ± 2.03
Melon	100 ± 1.87	80 ± 1.61	60 ± 1.64	20 ± 0.08	0 ± 0.01	100 ± 2.07	100 ± 2.81

**Table 3:** Toxicity testing of RBB dye against *Artemia salina*.

Sample	Concentration μg/mL	Initial Number	Average Mortality	Mortality %
RBB dye	10	15	8 ± 0.07	53.33
	100	15	13 ± 0.62	86.6
	1000	15	15 ± 1.46	100
Treated sample of SN40b	10	15	0 ± 0.02	0
	100	15	0 ± 0.07	0
	1000	15	1 ± 0.86	6.6
Treated sample of SN8c	10	15	0 ± 0.00	0
	100	15	0 ± 0.06	0
	1000	15	2 ± 1.01	13.33
Real Textile Effluents	10	15	10 ± 0.08	66.66
	100	15	14 ± 0.87	93.33
	1000	15	15 ± 1.06	100
Saline water (Control)		15	0 ± 0.00	0

## Data Availability

The data presented in this study are available on request from the corresponding author.
